# Alternating current magnetic susceptibility of a ferronematic

**DOI:** 10.3762/bjnano.8.251

**Published:** 2017-11-27

**Authors:** Natália Tomašovičová, Jozef Kováč, Veronika Gdovinová, Nándor Éber, Tibor Tóth-Katona, Jan Jadżyn, Peter Kopčanský

**Affiliations:** 1 Institute of Experimental Physics, Slovak Academy of Sciences, Watsonova 47, Košice, Slovakia; 2 Institute for Solid State Physics and Optics, Wigner Research Centre for Physics, Hungarian Academy of Sciences, H-1525, Budapest, P. O. Box 49, Hungary; 3 Institute of Molecular Physics, Polish Academy of Sciences, 60179, Poznan, Poland

**Keywords:** ac magnetic susceptibility, ferronematics, magnetic particles

## Abstract

We report on experimental studies focusing on the dynamic ac magnetic susceptibility of a ferronematic. It has been shown recently, that in the isotropic phase of a ferronematic, a weak dc bias magnetic field of a few oersteds increases the ac magnetic susceptibility. This increment vanishes irreversibly if the substance is cooled down to the nematic phase, but can be reinduced by applying the dc bias field again in the isotropic phase [Tomašovičová, N. et al. *Soft Matter ***2016**, *12,* 5780–5786]. The effect has no analogue in the neat host liquid crystal. Here, we demonstrate that by doubling the concentration of the magnetic nanoparticles, the range of the dc bias magnetic field to which the ferronematic is sensitive without saturation can be increased by about two orders of magnitude. This finding paves a way to application possibilities, such as low magnetic field sensors, or basic logical elements for information storage.

## Introduction

The great attention that nematic liquid crystals (LCs) have attracted in recent decades is due to the anisotropy of their physical properties. This anisotropy allows for a realignment of their director (the axis of cylindrical symmetry) by external electrical or magnetic fields, or by shear [1]. In common nematics, the threshold voltage of the reorientational response is just a few volts, owing to the relatively large anisotropy of the dielectric permittivity. Analogous effects exist with magnetic fields. However, the threshold magnetic fields are high (*B* = μ_0_*H* ≈ 1 T) as a consequence of the small diamagnetic susceptibility anisotropy (χ*_a_* ≈ 10^−6^) of liquid crystals. As a way of lowering the required applied magnetic field, doping liquid crystals with magnetic nanoparticles (MNPs) has been proposed [2]. The stable colloidal suspensions of MNPs in nematic LCs are now known as ferronematics (FNs). Following the first implementation of ferronematics [3], they soon have become favourite targets for research. Several theoretical models have been developed by extending the Ericksen–Leslie continuum theory [4–6], or the hydrodynamic description [1,7–8] for taking into account the interactions between MNPs, the host liquid crystal and the magnetic field. Experiments have proved that MNPs can alter the threshold fields for structural (e.g., Fréedericksz) transitions [9–11]; unexpected magneto-optical [12–13] and magneto-dielectric effects [14] have been found and shifts in the isotropic-to-nematic phase-transition temperature with and without magnetic field have been demonstrated [15–17]. For the interpretation of the latter effect mean-field theoretical models have been developed [18–19]. These studies have proven that doping modifies the material properties of the host liquid crystal. The improvement of certain properties, such as the capability of these materials to respond to external magnetic field more efficiently, comes from the collective properties of the MNPs dispersed in the liquid crystal. In this respect, investigation of such colloidal LC systems opens up new perspectives even for applications.

While the consequences of the behavior of FNs in static magnetic fields have been studied widely, much less is known about their dynamic properties. Theoretical calculations have been performed for various geometries to uncover how the magnetic field affects the flow properties of FNs [1,20], the pattern forming instabilities in FNs [8] or the behavior in rotating magnetic fields [21–25]. Experimental studies on dynamics are very scarce [26], thus most theoretical predictions still await experimental justification. In a recent work [27], we investigated the response of FNs to a small alternating magnetic field, measuring the ac magnetic susceptibility. We found that, unexpectedly, a small bias magnetic field (*H*_dc_ above ca. 10 Oe) applied to the ferronematic in the isotropic phase increased its ac susceptibility by about 10%. This increment vanished irreversibly at passing the isotropic-to-nematic phase transition on cooling (unless the bias field is applied again in the isotropic phase). A phenomenological explanation of the experimental results related this behavior to defect-mediated aggregation and magnetic-field-assisted disaggregation of MNPs [27].

In principle, the effect can provide a concept for potential future applications as sensors, or logical gates in micro- and nanodevices. However, the ferronematic composition investigated in [27] turned out to be sensitive to *H*_dc_ > 8 Oe only. Moreover, the effect of the increase in the ac susceptibility saturated for *H*_dc_ *>* 10 Oe. Therefore, the ferronematic composition reported in [27] can serve as a logical gate with a “yes” or “no” response to a prior existence of the biasing magnetic field *H*_dc_ *>* 8 Oe, rather than to function as a sensor that can sense and measure the magnitude of *H*_dc_.

The present work aims at the optimization of the ferronematic composition for sensor applications, through broadening the range of the magnetic field to which the suspension is sensitive without saturation.

## Experimental

Measurements were performed in a FN sample based on the calamitic thermotropic liquid crystal 4-*n*-hexyl-4'-cyanobiphenyl (6CB) [28–29]. This liquid-crystalline matrix was doped with spherical magnetic particles purchased from Ocean NanoTech. The mean diameter of the Fe_3_O_4_ magnetic particles was *d* = 20 nm. They were coated with oleic acid and dissolved in chloroform. This suspension was admixed to the liquid crystal, and the solvent was let to evaporate. The final volume concentration of the solid particles was 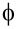 = 2 × 10^−4^, i.e., two times larger than in the FN investigated recently [27].

**Figure 1:**
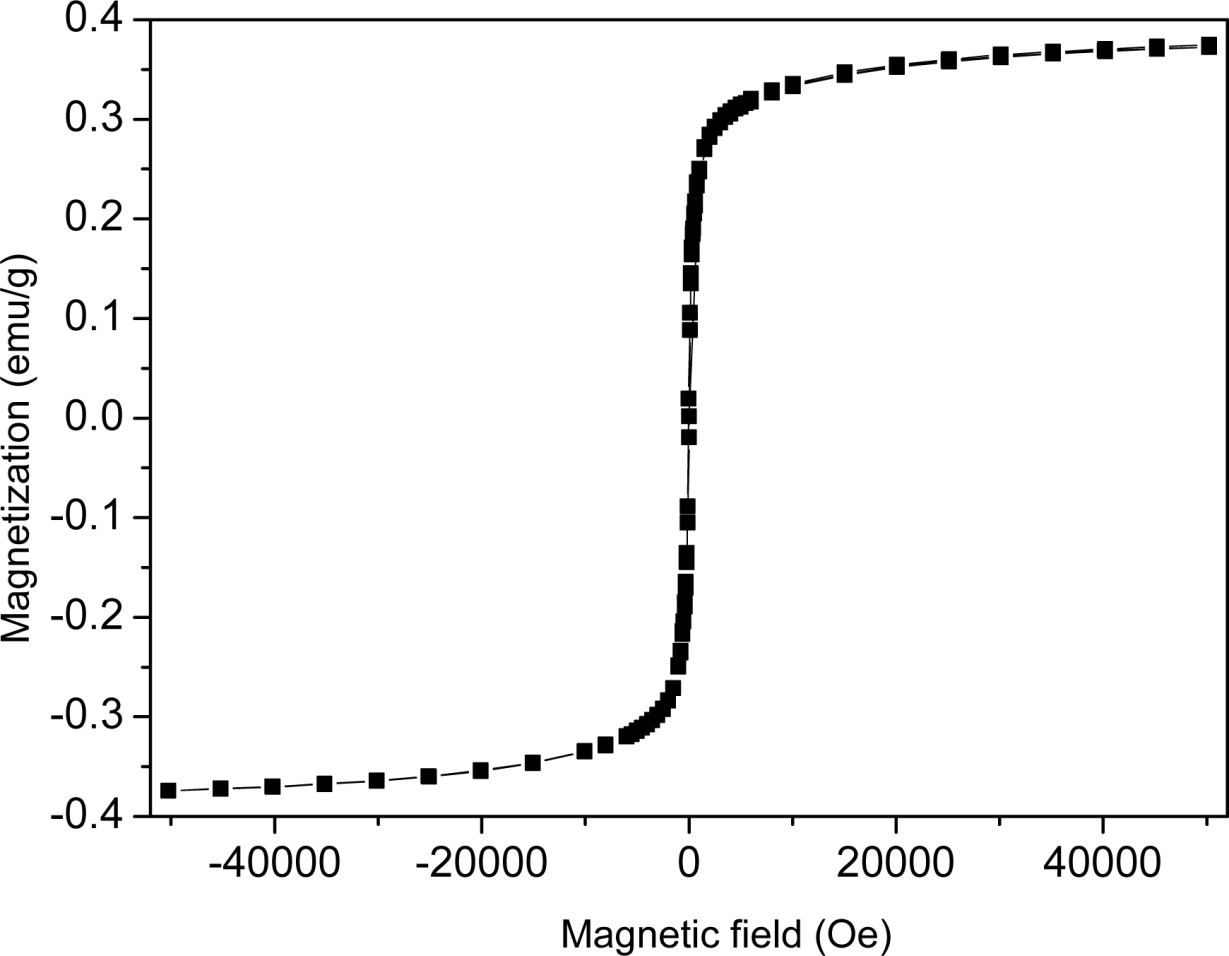
Magnetization curve of the powder of Fe_3_O_4_ magnetic nanoparticles, measured at 285 K.

For magnetic measurements the sample was filled into cylindrical capsules of 2.5 mm in diameter and 6.5 mm in length. The magnetic properties were measured with a SQUID magnetometer (Quantum Design MPMS 5XL) [30] in a magnetic field directed along the cylindrical axis of the capsules. Figure 1 shows the magnetization curve of the powder of MNPs obtained by evaporating the chloroform, measured at 285 K. This figure proves that the particles are superparamagnetic.

The temperature of the phase transition of the samples was detected by independent capacitance measurements in a capacitor made of ITO-coated glass electrodes (AWAT). The capacitor with an electrode area of approximately 5 mm × 5 mm was placed into a regulated thermostat system; the temperature was stabilized with an accuracy of 0.05 °C. The distance between the electrodes (sample thickness) was *D* = 5 μm. The capacitance was measured at a frequency of 1 kHz with an Andeen Hagerling high-precision capacitance bridge. The samples were first heated to the isotropic phase; then the measurement was carried out while cooling with a rate of 1 °C/min.

## Results and Discussion

**Figure 2:**
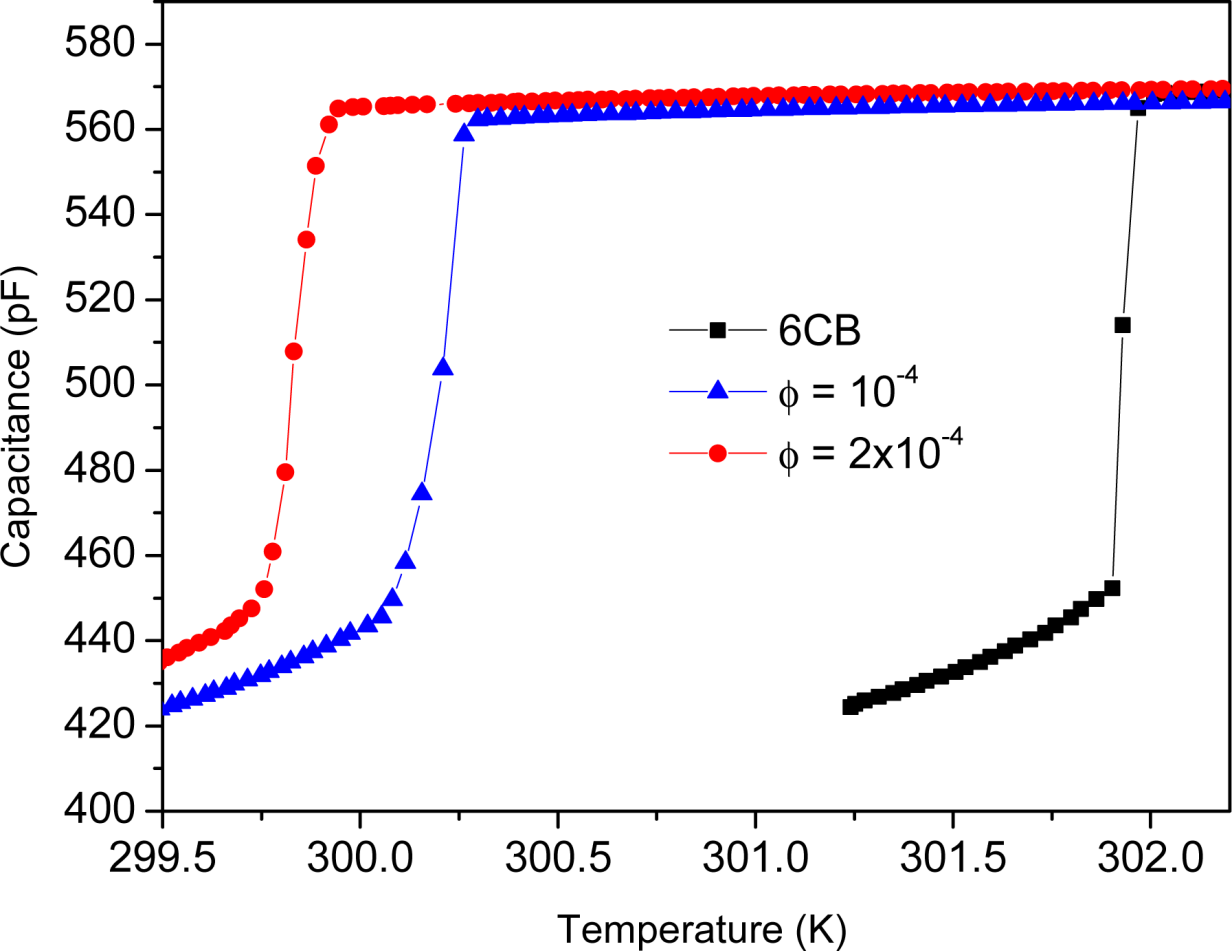
Temperature dependence of the capacitance of 6CB and of two ferronematic samples with different MNP concentrations, 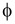.

The temperature *T*_IN_ of the phase transition from isotropic (I) to nematic (N) was determined by capacitance measurements presented in Figure 2. *T*_IN_ of neat 6CB is 302 K, while doping with the MNPs shifted the phase transition temperature to a lower value of *T*_IN_ ≈ 300 K. This shift in *T*_IN_ is slightly larger than that obtained at the lower dopant concentration of 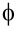 = 10^−4^ [27]).

**Figure 3:**
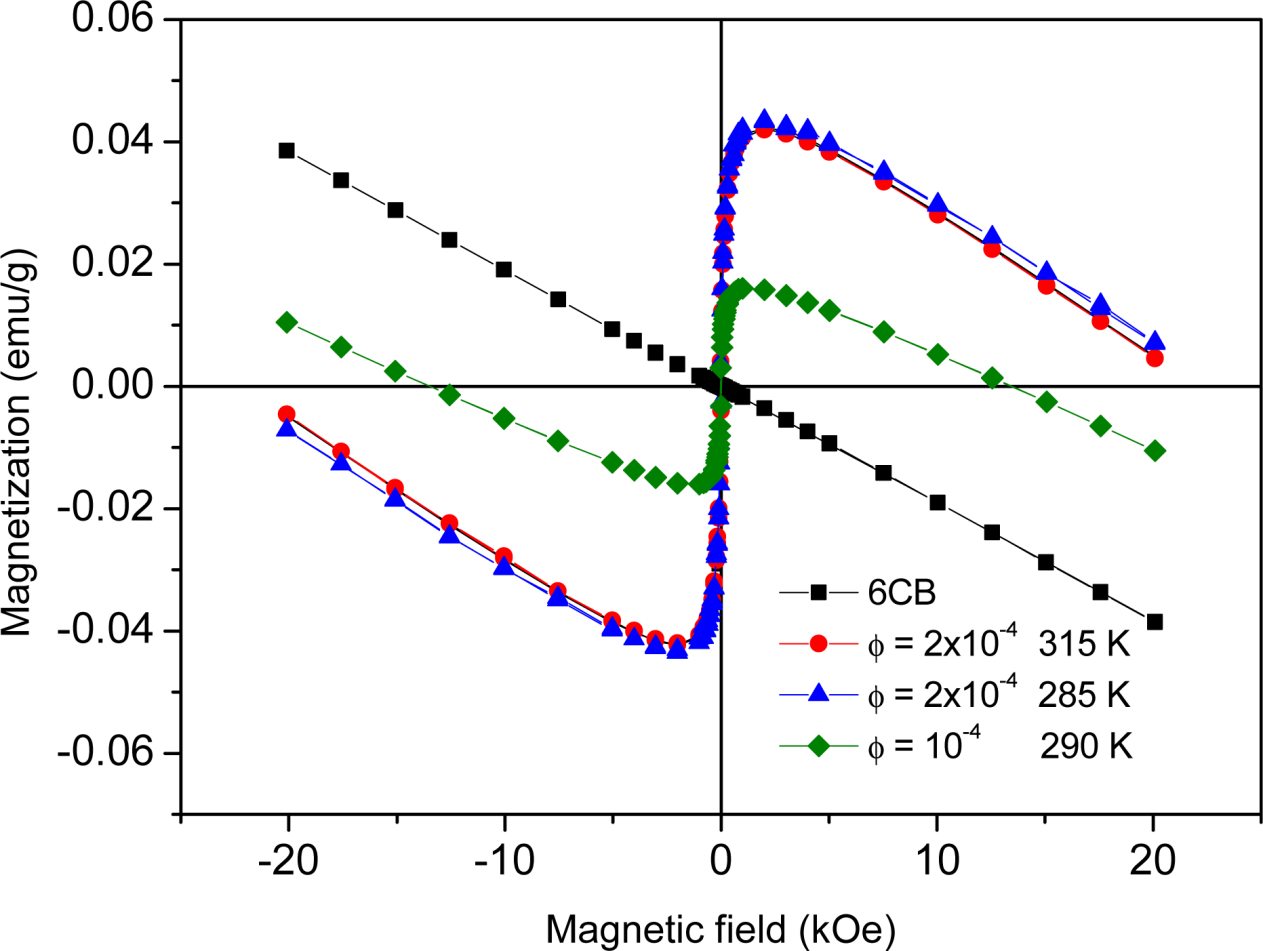
Magnetization curves of neat 6CB and the FN with a MNP concentration of 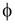 = 10^−4^ in the nematic phase, as well as of the FN with a higher concentration of MNPs (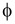 = 2 × 10^−4^ ) in the nematic (285 K) and isotropic (315 K) phases.

Figure 3 shows the magnetization curves of neat 6CB and of the two 6CB-based ferronematics (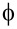 = 10^−4^ and 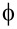 = 2 × 10^−4^), measured in the nematic phase, and that of the 6CB-based FN with a MNP concentration of 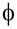 = 2 × 10^−4^, measured in the isotropic phase (*T* = 315 K). The straight line for 6CB indicates the usual diamagnetic behavior. In contrast to that, at low magnetic fields the FN composites are superparamagnets; they exhibit no hysteresis. At higher magnetic fields (*H >* 3000 Oe), diamagnetism of the host LC matrix becomes dominating. The distances between the magnetization curves with negative slope of 6CB and of the FNs at these high magnetic fields correspond to the saturation magnetization of the MNPs. For higher concentration of MNPs this distance is larger, as expected. No significant difference is found between the behavior in the isotropic and in the nematic phase, implying that the type of the fluid phase of the FN does not affect the quasi-static magnetic properties.

The ac magnetic susceptibility, χ, of the prepared sample was measured in the same experimental geometry as the magnetization curves. An ac magnetic field of 1 Oe was applied at a frequency of *f* = 10 Hz. To measure the temperature dependence of χ, the sample was first heated to 315 K (isotropic phase), then slowly cooled down to the nematic phase and finally slowly heated up again to 315 K. The ac susceptibility was measured at a sequence of temperatures (with a temperature step size of 1 K). At each temperature the sample was thermally stabilized for 3 min before determining χ. Then the next temperature was achieved with a heating/cooling rate of 0.5 K/min. Before each cooling–heating cycle, first a dc magnetic field *H*_dc_ was applied in the isotropic phase typically for 10 min, then it was switched off. This is the same protocol as that used in [27].

**Figure 4:**
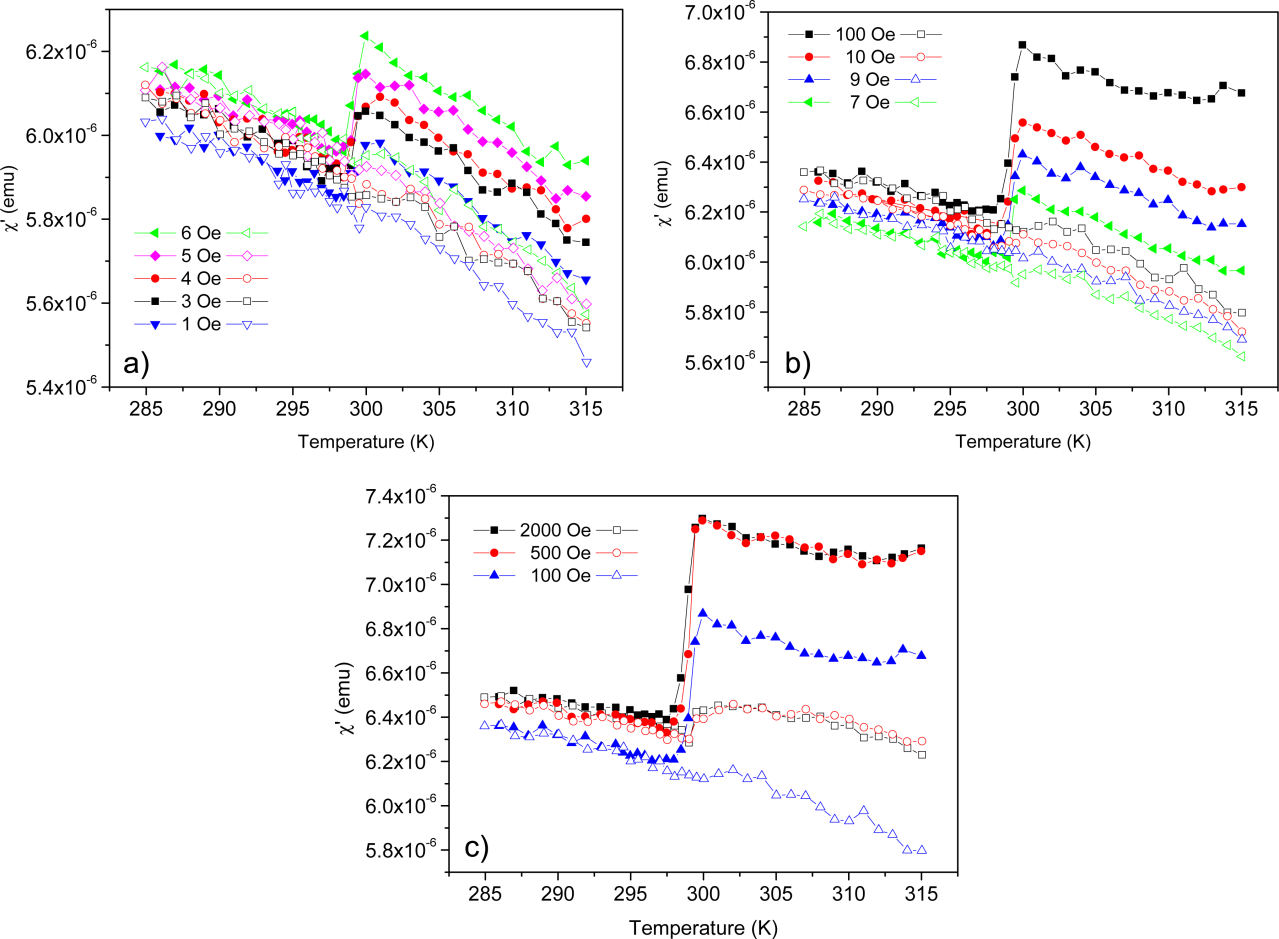
Temperature dependence of the real part χ′ of the ac susceptibility of the 6CB-based FN, measured in a cooling–heating cycle, after applying and switching off a dc magnetic field *H*_dc_ of (a) 1–6 Oe, (b) 7–100 Oe and (c) 100–2000 Oe. Solid symbols stand for cooling, while open symbols stand for heating.

Figure 4 presents the real part χ′ of the ac magnetic susceptibility as a function of temperature for different *H*_dc_ ranging from 1 to 2000 Oe, during cooling (solid symbols) as well as during heating (open symbols). It can be seen that χ′(*T*) has a negative slope in the isotropic, as well as in the nematic phase, except in a very narrow temperature range during cooling, where χ′ undergoes a sudden reduction of up to about 10%. Passing the same temperature range in heating, typically, does not affect χ′. It changes monotonically without any jump. The temperature range where the jump in χ′ occurs on cooling corresponds to the temperature of the isotropic-to-nematic phase transition *T*_IN_, cf. Figure 4 and Figure 2.

**Figure 5:**
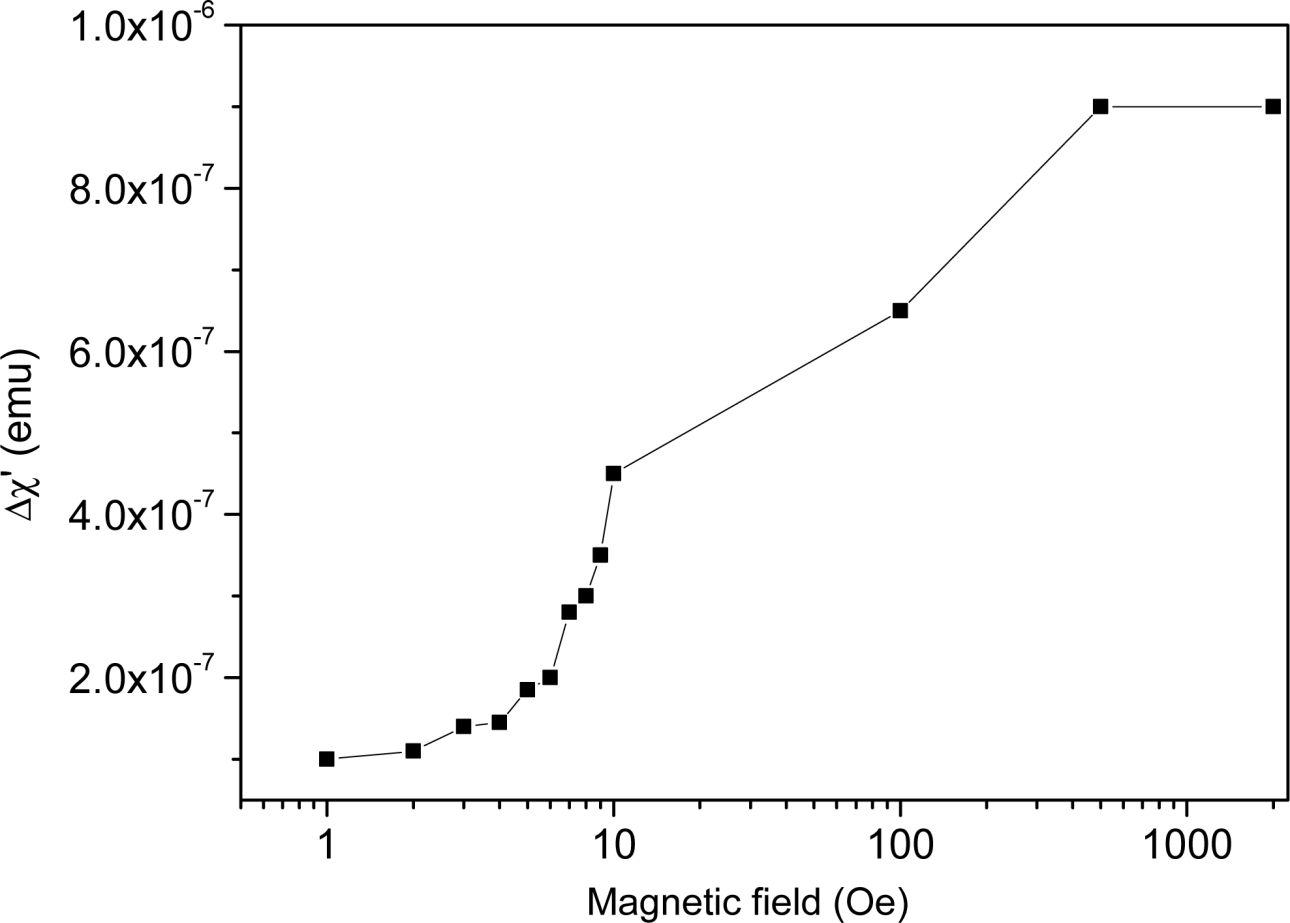
Magnitude of the reduction in the ac susceptibility Δχ′ at the isotropic-to-nematic phase transition as a function of the dc bias magnetic field.

Figure 4 clearly shows that the ac magnetic susceptibility of the FN is a two-valued function of the temperature in the isotropic phase. The lower value belongs to the ferronematic, which has not yet been subjected to a dc magnetic field after heating up to the isotropic phase. The other (higher) value is induced by the application of a relatively small dc magnetic field *H*_dc_. The magnitude Δχ′ of the sudden reduction in χ′ depends on *H*_dc_, as shown in Figure 5. It saturates at approximately 500 Oe.

The increment Δχ′ is evidently due to the presence of magnetic nanoparticles, since this effect is completely absent in the neat liquid crystal [27]. According to our model [27], *H*_dc_ aligns the magnetic moments of nanoparticle aggregates, forcing them into a state with inconveniently high energy, thus promoting the breakaway of single particles from the aggregates. This increases the number of particles capable for magnetic reorientation by the ac testing magnetic field (large aggregates practically do not contribute to χ′). These changes in the particle distribution persist in the isotropic phase, but are annulled at entering into the nematic phase, since the disclinations emerging at the isotropic-to-nematic phase transition strongly promote particle aggregation.

Analyzing the above presented results, and comparing them with those reported in [27] for the FN with a lower concentration of MNPs, several conclusions can be drawn:

**(i.)** Most importantly, the range of the bias magnetic field to which the FN is sensitive without saturation has increased by about two orders of magnitude with the increase of the concentration of MNPs, 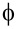. Namely, for 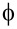 = 10^−4^ this range was found between 8 Oe *< H*_dc_ ≤ 10 Oe [27], while for 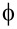 = 2 × 10 ^−4^ it is 1 Oe ≤ *H*_dc_ ≤ 500 Oe (Figure 5).

**(ii.)** The highest achievable relative magnitude Δχ′/χ′ of the sudden drop in the ac susceptibility at *T*_IN_ seems to be independent of the concentration of MNPs: It is about 10% for both 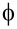 = 10^−4^ [27] and 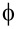 = 2 × 10^−4^. At higher *H*_dc_ the effect saturates for both concentrations, and the relative magnitude of the drop remains the same. It is a question for future research whether this holds for FNs of different compositions (different host LC and/or different MNPs).

**(iii.)** For the higher concentration of MNPs a higher value of ac susceptibility χ′ is observed (cf. Figure 4 and Figure 4 of [27]), which is in line with our phenomenological model: The higher concentration of MNPs results in a larger number of individual particles and dimers that contribute to the ac susceptibility. Nevertheless, the effect is not linear; multiparticle aggregates with nearly closed flux virtually do not contribute to χ′ and at the higher concentration there is an enhanced tendency for the formation of such aggregates.

**(iv.)** The value of the ac susceptibility in Figure 4 is temperature-dependent, while no such dependence was found for the lower concentration of 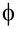 = 10^−4^ (see Figures 3–5 of [27]). The fact the χ′ is independent of the temperature at the lower concentration is surprising. Namely, although diamagnetism is temperature-independent in the temperature range of our measurements, ferromagnetism decreases with temperature as *T^−3/2^*. The decrease in spontaneous magnetization at higher temperatures is caused by the increased excitation of spin waves. The decrease of the ac susceptibility with the temperature is related to the decrease of magnetization. Therefore, the absence of a temperature dependence of χ′ at the lower concentration of MNPs [27] is a strong indication of the absence of interaction between MNPs.

**(v.)** The value of the ac susceptibility in the nematic phase, as well as in the isotropic phase during heating, slightly increases with the increase of *H*_dc_ (this effect was again unnoticed at the lower concentration of MNPs [27]). This behavior indicates that a larger *H*_dc_ has a slightly higher disaggregation capability, i.e., it produces a slightly larger number of single MNPs or dimers contributing to χ′(*T*), and some of them remain nonaggregated above the isotropic-to-nematic phase transition temperature.

**(vi.)** In addition to the bias-field-dependent shift of the χ′(*T*) curve mentioned above, for *H*_dc_ ≥ 500 Oe, where the magnitude of the sudden drop Δχ′ saturates, the value of χ′ slightly increases during heating at the nematic-to-isotropic phase transition temperature. This effect is still not fully understood, and will be a subject for future studies, together with the more detailed analysis of the processes leading to the saturation of Δχ′ (Figure 5).

In summary, we have shown experimentally that the sensitivity range of FNs to magnetic fields can be extended significantly by the optimization of the FN composition, and that, in principle, the effect may be used for sensing low magnetic fields.

## References

[1] Jarkova E, Pleiner H, Müller H-W, Brand H R (2003). J Chem Phys.

[2] Brochard F, de Gennes P G (1970). J Phys (Paris).

[3] Chen S-H, Amer N M (1983). Phys Rev Lett.

[4] Burylov S V, Raikher Y L (1995). Mater Sci Eng, C.

[5] Burylov S V, Raikher Y L (1995). Mol Cryst Liq Cryst.

[6] Zakhlevnykh A N, Petrov D A (2016). J Magn Magn Mater.

[7] Jarkova E, Pleiner H, Müller H-W, Fink A, Brand H R (2001). Eur Phys J E.

[8] Ryskin A B, Pleiner H, Müller H W (2003). Eur Phys J E.

[9] Tomašovičová N, Koneracká M, Kopčanský P, Timko M, Závišová V, Jadzyn J (2006). Phase Transitions.

[10] Kopčanský P, Tomašovičová N, Koneracká M, Závišová V, Timko M, Džarová A, Šprincová A, Éber N, Fodor-Csorba K, Tóth-Katona T (2008). Phys Rev E.

[11] Kopčanský P, Tomašovičová N, Koneracká M, Timko M, Závišová V, Éber N, Fodor-Csorba K, Tóth-Katona T, Vajda A, Jadzyn J (2010). J Magn Magn Mater.

[12] Podoliak N, Buchnev O, Buluy O, D’Alessandro G, Kaczmarek M, Reznikov Y, Sluckin T J (2011). Soft Matter.

[13] Buluy O, Nepijko S, Reshetnyak V, Ouskova E, Zadorozhnii V, Leonhardt A, Ritschel M, Schönhense G, Reznikov Y (2011). Soft Matter.

[14] Tomašovičová N, Timko M, Mitróová Z, Koneracká M, Rajňak M, Éber N, Tóth-Katona T, Chaud X, Jadzyn J, Kopčanský P (2013). Phys Rev E.

[15] Martinez-Miranda L J, Kurihara L K (2009). J Appl Phys.

[16] Kopčanský P, Tomašovičová N, Koneracká M, Závišová V, Timko M, Hnatič M, Éber N, Tóth-Katona T, Jadzyn J, Honkonen J (2011). IEEE Trans Magn.

[17] Gdovinová V, Tomašovičová N, Éber N, Tóth-Katona T, Závišová V, Timko M, Kopčanský P (2014). Liq Cryst.

[18] Gorkunov M V, Osipov M A (2011). Soft Matter.

[19] Raikher Y L, Stepanov V I, Zakhlevnykh A N (2013). Soft Matter.

[20] Makarov D V, Zakhlevnykh A N (2008). J Magn Magn Mater.

[21] Boychuk A N, Zakhlevnykh A N, Makarov D V (2015). J Exp Theor Phys.

[22] Boychuk A N, Makarov D V, Zakhlevnykh A N (2016). Eur Phys J E.

[23] Boychuk A N, Makarov D V, Zakhlevnykh A N (2017). J Mol Liq.

[24] Zakhlevnykh A N, Lubnin M S, Petrov D A (2017). J Magn Magn Mater.

[25] Shoarinejad S, Ghazavi M (2017). Soft Mater.

[26] Potisk T, Svenšek D, Brand H R, Pleiner H, Lisjak D, Osterman N, Mertelj A (2017). Phys Rev Lett.

[27] Tomašovičová N, Kováč J, Raikher Y, Éber N, Tóth-Katona T, Gdovinová V, Jadzyn J, Pinčák R, Kopčanský P (2016). Soft Matter.

[28] Gray G W, Harrison K J, Nash J A (1973). Electron Lett.

[29] Czechovski G, Czerkas S, Jadżyn J (2001). Z Naturforsch, A.

[30] (2017). Fundamentals of Magnetism and Magnetic Measurements. Featuring Quantum Design’s Magnetic Properties Measurement System.

